# Complete mitochondrial genome of *Hydrellia griseola* (Diptera, Ephydridae)

**DOI:** 10.1080/23802359.2017.1372703

**Published:** 2017-09-01

**Authors:** Liang Wang, Ding Yang, Shuangmei Ding, Xin Li, Junhua Zhang, Mei Tao

**Affiliations:** a College of Plant Protection, Yunnan Agricultural University, Kunming, China;; b College of Plant Protection, China Agricultural University, Beijing, China;; c Institute of Plant Quarantine, Chinese Academy of Inspection and Quarantine, Beijing, China

**Keywords:** Ephydridae, phylogeny, mitogenome, *Hydrellia griseola*

## Abstract

The complete mitochondrial genome (mitogenome) of *Hydrellia griseola* has been reported in this study. This is the first sequenced complete mitogenome of the family Ephydridae. The complete mitogenome is 16,159 bp in length, including 13 protein-coding genes, two ribosomal RNAs, 22 transfer RNAs, and a partial sequence of the AT-rich region, and the AT-rich region contains several characteristic repeated sequences. In addition, the nucleotide composition of the coding region was 38.7% of A, 37.0% of T, 14.2% of C, 10.1% of G, 75.7% of A + T content. Four complete mitochondrial genome data of related species are download from GenBank, and all of them are used in Neighbor-Join analyses. The result consistently supports the monophyly of Ephydroidea.

The Ephydroidea is one of the most surely grounded monophyletic groups in Acalyptratae (Hennig 1958, 1971; Griffiths 1972). It is divided into five families, Curtonotidae, Camillidae, Drosophilidae, Diastatidae, and Ephydridae (McAlpine 1989). Ephydridae is a highly diversified group with 129 genera and about 2000 known species all over the world, and 55 genera and about 200 known species in China. Feeding habits of their adults are varied and known for only a small proportion of species (Deyrup et al. 2008). Most adults consume algae or bacterial slurries, but some are predators on smaller arthropods, scavengers, or nectar feeders (Wirth et al. 1987). Up to now, there are just some partial sequences in GenBank database, but the complete mitochondrial genome data are not available. Complete mitochondrial genomes of its relative species, *Pachycerina decemlineata, Bactrocera cucurbitae, Nemopoda mamaevi, Drosophila melanogaster* are available on internet. Here, we sequenced the complete mitochondrial genome of *Hydrellia griseola* (Fallén, 1813), the first representative of family Ephydridae for the further research.

Specimens of *Hydrellia griseola* were collected in Lianhua Mountain, Kangle County, Gansu Province, China and also identified by Junhua Zhang. Specimens are deposited in the Entomological Museum of China Agricultural University, Beijing.

The genomic DNA was extracted from adult’s muscle tissues of the thorax and legs using the DNeasy DNA Extraction kit (TIANGEN, Beijing, China), and sequenced under the next generation sequence technology.

The complete mitochondrial genome of *Hydrellia griseola* contains 22 transfer RNA genes, 13 protein-coding genes (PCGs), two ribosomal RNA genes and non-coding control region, which were similar with related reports before (Kang et al. 2014; Li et al. 2016; Wang, Ding, et al. 2016; Wang, Liu, et al. 2016; Wang, Wang, et al. 2016).

The completed mitochondrial genome of *Hydrellia griseola* is 16,159 bp in length, and the nucleotide composition of coding region was 38.7% of A, 37.0% of T, 14.2% of C, 10.1% of G, 75.7% of A + T content. The codon ATG was the most popular start codon shared with ATP6, COX2, COX3, CYTB, ND4, ND4L, and start codon ATT was shared with ATP8, ND2, ND3, ND5, and ND6. Particularly, the COX1 begins with codon ACG, and the ND1 begins with codon TTG. The conservative stop codon TAA was shared with ATP6, ATP8, COX3, ND2, ND4L, ND6, the stop codon T was shared with COX1, COX2, ND4, ND5, while the gene CYTB, ND3 was ended with stop codon TAG, and the ND1 was ended with stop codon TA.

Based on 13 PCGs data among seven species including *Simulium variegatum* from Simulidae and *Anopheles oryzalimnetes* from Culicidae as outgroups, we conducted a phylogenetic analysis employed the Neighbor-Join (NJ) method implemented in Mega 7 (Kumar et al. 2016). As a result (Figure 1), the monophyly of Ephydroidea were consistently supported.

**Figure 1.**
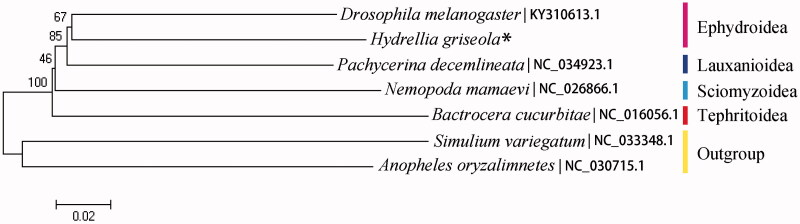
Phylogenetic tree among seven species which consist of five Acalyptratae species and two outgroups including Simulidae and Culicidae (*data sequenced in this study).
